# Phenolic Glycolipid Facilitates Mycobacterial Escape from Microbicidal Tissue-Resident Macrophages

**DOI:** 10.1016/j.immuni.2017.08.003

**Published:** 2017-09-19

**Authors:** C.J. Cambier, Seónadh M. O’Leary, Mary P. O’Sullivan, Joseph Keane, Lalita Ramakrishnan

**Affiliations:** 1 Department of Immunology, University of Washington, Seattle, WA 98195, USA; 2 Molecular Immunity Unit, Department of Medicine, University of Cambridge, MRC Laboratory of Molecular Biology, Cambridge CB2 0QH, UK; 3 Department of Chemistry, Stanford University, Stanford, CT 94305, USA; 4 Department of Clinical Medicine, Trinity Translational Medicine Institute, Trinity College Dublin, Dublin 8, Ireland; 5 Department of Microbiology, University of Washington, Seattle, WA 98195, USA; 6 Department of Medicine, University of Washington, Seattle, WA 98195, USA

## Abstract

*Mycobacterium tuberculosis* (Mtb) enters the host in aerosol droplets deposited in lung alveoli, where the bacteria first encounter lung-resident alveolar macrophages. We studied the earliest mycobacterium-macrophage interactions in the optically transparent zebrafish. First-responding resident macrophages phagocytosed and eradicated infecting mycobacteria, suggesting that to establish a successful infection, mycobacteria must escape out of the initially infected resident macrophage into growth-permissive monocytes. We defined a critical role for mycobacterial membrane phenolic glycolipid (PGL) in engineering this transition. PGL activated the STING cytosolic sensing pathway in resident macrophages, inducing the production of the chemokine CCL2, which in turn recruited circulating CCR2^+^ monocytes toward infection. Transient fusion of infected macrophages with CCR2^+^ monocytes enabled bacterial transfer and subsequent dissemination, and interrupting this transfer so as to prolong mycobacterial sojourn in resident macrophages promoted clearing of infection. Human alveolar macrophages produced CCL2 in a PGL-dependent fashion following infection, arguing for the potential of PGL-blocking interventions or PGL-targeting vaccine strategies in the prevention of tuberculosis.

**Video Abstract:**

## Highlights

• Microbicidal tissue-resident macrophages are first responders to mycobacteria
• Mycobacterial phenolic glycolipid induces macrophage CCL2 through STING activation
• CCL2 recruits mycobacterium-permissive monocytes to the tissue-resident macrophage
• Mycobacteria transfer from tissue macrophage to monocyte through a cell fusion event

## Graphical Abstract

**Figure:**
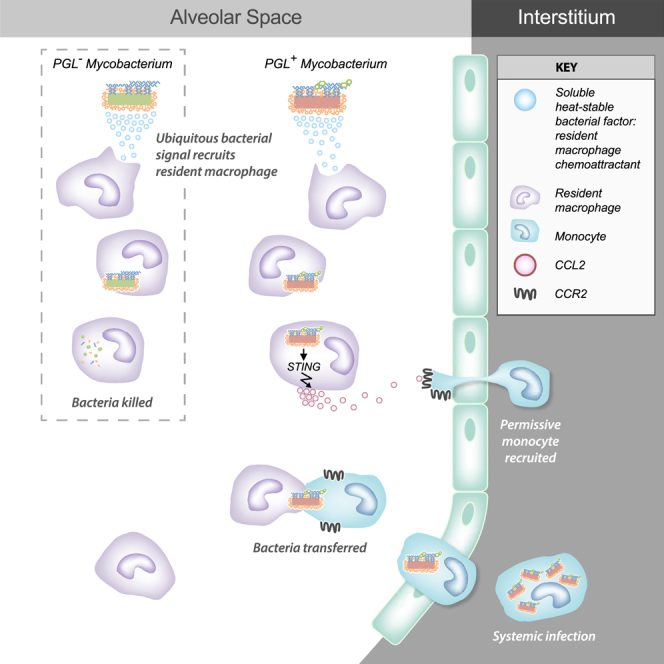

## Teaser

Cambier et al. find that activation of the STING pathway in lung-resident microbicidal macrophages by the mycobacterial surface lipid PGL enables bacterial escape by inducing the recruitment of mycobacterium-permissive monocytes via the CCL2-CCR2 chemokine axis. Their findings reveal a relocation strategy that enables mycobacterial dissemination, and argue for the potential of interventions targeting PGL in the prevention of tuberculosis.

## Introduction

When *M. tuberculosis* (Mtb) is aerosolized into the lower lung, it first encounters lung-resident alveolar macrophages that patrol the air-lung epithelium interface (Srivastava et al., 2014). In the first few days post-infection, Mtb is found exclusively within alveolar macrophages (Srivastava et al., 2014, Urdahl, 2014, Wolf et al., 2007). Thereafter, it traverses the lung epithelium to reside within other myeloid cells that have aggregated into granulomas (Cambier et al., 2014a, Srivastava et al., 2014). The difficulty of tracking the early fate of individual mycobacteria in traditional animal models has precluded elucidation of how mycobacteria move from alveolar macrophages into other cells and indeed how they survive these broadly microbicidal first responders (Hocking and Golde, 1979).

We have exploited the optical transparency of the zebrafish larva to study the early mycobacterium-phagocyte interactions by infecting *Mycobacterium marinum* (Mm), a close genetic relative of Mtb, into the zebrafish larval hindbrain ventricle, an epithelium-lined cavity (Cambier et al., 2014b, Yang et al., 2012). In this model, pathogenic mycobacteria manipulate host responses immediately upon infection so as to inhibit the recruitment of neutrophils and microbicidal monocytes, and instead recruit and infect mycobacterium-permissive myeloid cells (Cambier et al., 2014b, Yang et al., 2012). To avoid detection by microbicidal monocytes, mycobacteria mask exposed pathogen-associated molecular patterns (PAMPs) with the cell-surface phthiocerol dimycoceroserate (PDIM) lipid, thus preventing recognition of PAMPs by Toll-like receptors (TLRs) (Cambier et al., 2014b). Mycobacteria thus inhibit monocyte signaling through TLRs, which would normally recruit prototypical microbicidal iNOS-expressing monocytes. In conjunction, pathogenic mycobacteria recruit growth-permissive monocytes using a PDIM-related surface lipid, phenolic glycolipid (PGL) that induces the host monocyte chemokine CCL2. CCL2 recruits mycobacterium growth-permissive monocytes through signaling via its cognate receptor CCR2. The recruitment of growth-permissive monocytes is critically important for the ability of mycobacteria to establish infection. PGL-deficient mycobacteria fail to recruit normal numbers of monocytes and their ability to establish infection is attenuated (Cambier et al., 2014b).

However, mycobacteria still have to contend with resident macrophages that are thought to be the first phagocytes encountered during infection (Srivastava et al., 2014). Here we found that resident macrophages are default first-responders to invading bacteria, including mycobacteria, and phagocytosed them rapidly. These first-responding resident macrophages were microbicidal to virulent mycobacteria, and capable of eradicating infection unless the mycobacteria escaped into more permissive cells. We found that PGL rapidly induces the production of CCL2 in the resident macrophages via a Sting-associated pathway. CCL2 recruited CCR2^+^ monocytes to the close proximity of the infected resident macrophage. The bacteria then transferred from microbicidal resident macrophages into these monocytes, thus escaping into a growth-permissive niche to establish infection. Resident macrophage-mycobacterium interaction is thus possibly the earliest determinant of whether infection will be established or cleared, with PGL acting as a very early mycobacterial immune evasion determinant. Furthermore, our findings suggest that STING and CCL2 are host susceptibility factors that act at the very first steps of infection.

## Results

### Resident Macrophages Are First Responders to Mm and Mucosal Commensal Pathogens through Sensing a Common Secreted Signal

**Figure 1:**
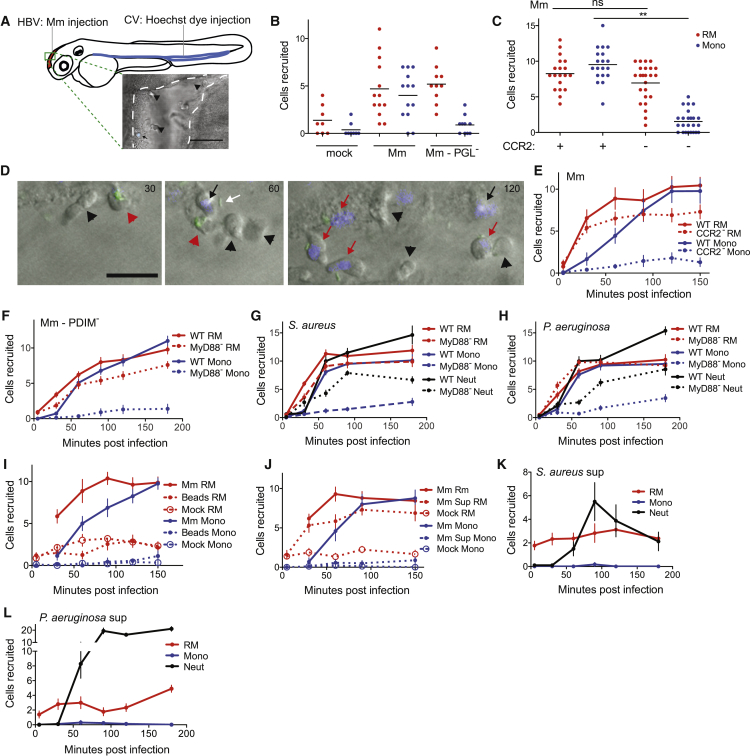
Resident Macrophages Are First Responders to Bacterial Infection (A) Cartoon of a 2 day post-fertilization (dpf) zebrafish showing the caudal vein (CV) and hindbrain ventricle (HBV) injection sites and representative image of HBV (outlined with white dashed line) with Hoechst dye negative resident macrophages (black arrowheads) and Hoechst dye positive monocyte (black arrow). Scale bar, 100 μm. (B) Mean resident macrophage (RM) and monocyte (Mono) recruitment at 3 hr post infection (hpi) into the HBV after infection with 80 wild-type Mm (Mm) or PGL-deficient Mm (Mm-PGL^−^). Significance testing done using one-way ANOVA, with Bonferroni’s post-test against mock injections. ^∗∗^p < 0.01. (C) Mean resident macrophage and monocyte recruitment at 3 hpi into the HBV of wild-type or Ccr2-deficient fish after infection with 80 wild-type Mm. Significance testing done using one-way ANOVA, with Bonferroni’s post-test for comparisons shown. ^∗∗^p < 0.01. (D) Representative images of uninfected resident macrophages (black arrowheads), uninfected monocytes (black arrows), infected resident macrophages (red arrowheads), infected monocytes (red arrows), and extracellular bacteria (white arrow) following infection of wild-type fish in the HBV with 80 wild-type green fluorescent Mm at 30, 60, and 120 min post infection (mpi). Scale bar, 20μm. (E) Mean resident macrophage and monocyte recruitment from 5 to 150 mpi in the HBV of wild-type or Ccr2-deficient fish after infection with 80 wild-type Mm. (F) Mean resident macrophage and monocyte recruitment from 5 to 180 mpi in the HBV of wild-type or Myd88-deficient fish after infection with 80 PDIM-deficient Mm (Mm – PDIM^−^). (G and H) Mean resident macrophage, monocyte, and neutrophil (Neut) recruitment from 5 to 180 mpi in the HBV of wild-type or Myd88-deficient fish following infection with 138 *S. aureus* (G) or 156 *P. aeruginosa* (H). (I) Mean resident macrophage and monocyte recruitment from 5 to 150mpi in the HBV of wild-type fish after injection with 80 wild-type Mm, 300 sterile beads, or mock injection. (J) Mean resident macrophage and monocyte recruitment from 5 to 150 mpi in the HBV of wild-type fish after infection with 80 wild-type Mm, an equivalent volume of wild-type Mm supernatant (Sup), or media mock. (K and L) Mean resident macrophage, monocyte, and neutrophil recruitment from 5 to 180 mpi in the HBV of wild-type fish after infection with *S. aureus* supernatant (K) or *P. aeruginosa* supernatant (L). (A – L) Results representative of at least three independent experiments.

When Mtb is aerosolized into mouse lung, it is found for the first few days exclusively within alveolar macrophages (Srivastava et al., 2014, Urdahl, 2014, Wolf et al., 2007). In the zebrafish larva, directly posterior to the hindbrain ventricle infection site (Figure 1A), is the brain which, like most organs, has a population of resident macrophages (Herbomel et al., 2001). We asked whether these brain-resident macrophages or microglia, analogous to the resident macrophages of the mammalian lung, participated in the immune response to mycobacterial infection. In addition to their tissue-specific functions, tissue-resident macrophages, including those of the brain, play a central role in host defense against infection (Casano and Peri, 2015). Like lung-resident macrophages, brain-resident macrophages phagocytose Mtb and produce inflammatory cytokines in response to it (Curto et al., 2004, Spanos et al., 2015).

To distinguish between brain-resident macrophages and monocytes, we used the nuclear dye Hoechst 33342 that does not cross the blood brain barrier; injection of Hoechst 33342 into the caudal vein of zebrafish larvae labels cells, including myeloid cells, in the body but not in the brain (Davis and Ramakrishnan, 2009). We injected Hoechst dye into the caudal vein and then injected wild-type Mm into the HBV 2 hr later (Figure 1A). Three hours following infection, recruited cells were identified as either brain-resident macrophages (Hoechst-negative) or peripheral monocytes (Hoechst-positive) (Figure 1A).

Our prior work had shown that myeloid cell recruitment to the HBV was substantially dependent on bacterial PGL and host Ccl2-Ccr2 (Cambier et al., 2014b). We asked whether recruitment of resident macrophages, monocytes, or both were dependent on these. Wild-type Mm recruited both resident macrophages and monocytes, whereas the PGL-deficient Mm strain (Δ*pks15*) recruited resident macrophages but not monocytes (Figure 1B). Correspondingly in Ccr2-deficient animals, wild-type Mm recruited resident macrophages but not monocytes (Figure 1C). We asked whether resident macrophages in the zebrafish larvae arrived more rapidly to mycobacteria, similar to resident macrophages in the mammalian lung. A temporal analysis revealed that they were the first responders to infection and arrived independently of Ccr2 signaling (Figures 1D and 1E). In contrast, monocytes arrived later and in a Ccr2-dependent fashion (Figures 1D and 1E). Thus, similar to Mtb infection of the mammalian lung, Mm infection of the zebrafish HBV recruits both resident macrophages and peripheral monocytes. The two cell types appear to be recruited sequentially, and through distinct pathways—Ccr2-independent for resident macrophages and Ccr2-dependent for peripheral monocytes.

We found that resident macrophages were also the first-responders in bacterial infections wherein overall myeloid cell recruitment is dependent on Toll-like receptor (TLR-MyD88) signaling rather than the CCL2-CCR2 axis (Cambier et al., 2014b), such as in the case of PDIM-deficient Mm (Δ*mmpL7*) and the mucosal commensal-pathogens *Staphylococcus aureus* and *Pseudomonas aeruginosa* (Figures 1F–1H). In addition to mononuclear phagocytes, *S. aureus* and *P. aeruginosa* elicited the early recruitment of neutrophils, which were distinguished from monocytes and macrophages using the transgenic *lyz::EGFP* zebrafish (Yang et al., 2012), through TLR-Myd88 signaling (Figures 1G and 1H) (Cambier et al., 2014b, Yang et al., 2012). In all cases, resident macrophage recruitment was independent of TLR-Myd88 signaling, as they were still responding toward infection in Myd88-deficient fish (Figures 1F–1H). Thus, tissue-resident macrophages appear to be default first-responders to invading bacteria, even those that elicit a robust protective neutrophilic response, with their recruitment to bacteria being independent of the TLR-Myd88 pathway.

We ruled out the possibility that mechanosensing of a foreign body at the infection site was driving resident macrophage recruitment (Wang et al., 2009) by showing that neither resident macrophages nor monocytes were recruited to sterile beads (Figure 1I). To examine whether resident macrophage recruitment is mediated by bacterial signals, we assayed recruitment of resident macrophages to supernatants of cultures of Mm, *S. aureus*, and *P. aeruginosa*; supernatants from these bacterial cultures recruited resident macrophages (and in the case of the latter two, neutrophils) but not monocytes (Figures 1J–1L). Thus, tissue-resident macrophages are recruited in response to a secreted factor(s) produced by both Gram+ and Gram− bacteria as well as mycobacteria.

### Mycobacteria Infection Elicits CCL2 Production in Resident Macrophages to Recruit Monocytes

**Figure 2:**
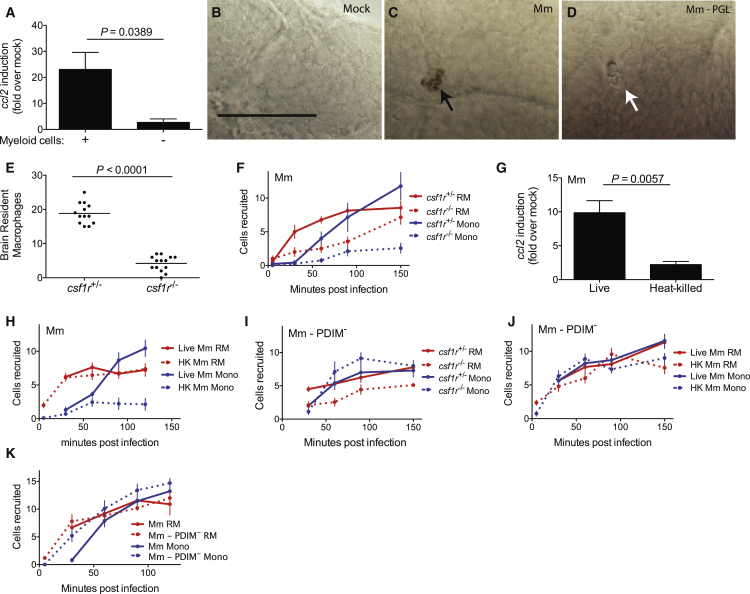
Mycobacteria Mediate CCR2-Dependent Monocyte Recruitment by Actively Inducing CCL2 in Resident Macrophages (A) *ccl2* messenger RNA levels (mean ± SEM of three biological replicates) induced at 3 hr after caudal vein infection of 2 dpf wild-type or myeloid cell-deficient fish with 250–300 wild-type Mm. (B–D) In situ hybridizations against zebrafish *ccl2* mRNA following hindbrain ventricle infections with vehicle (bacterial media) (B), 80 wild-type Mm (C), 80 Mm - PGL^−^ (D). Black arrows, *ccl2* mRNA-positive phagocytes; white arrows *ccl2* mRNA-negative phagocytes. Scale bar, 50μm. Results representative of three independent experiments. (E) Mean brain resident macrophage numbers of *csfr1*^+/−^ and *csfr1*^−/−^ zebrafish at 2dpf. Results representative of two independent experiments. (F) Mean resident macrophage and monocyte recruitment from 5 to 150 mpi in the HBV of *csfr1*^+/−^ or *csfr1*^−/−^ fish after infection with 80 wild-type Mm. (G) *ccl2* messenger RNA levels (mean ± SEM of three biological replicates) induced at 3 hr after caudal vein infection of 2 dpf wild-type fish with 250–300 live or heat-killed wild-type Mm. (H) Mean resident macrophage and monocyte recruitment from 5 to 120 mpi in the HBV of wild-type fish after infection with 80 live or heat-killed (HK) wild-type Mm. (I) Mean resident macrophage and monocyte recruitment from 5 to 150 mpi in the HBV of *csfr1*^+/−^ or *csfr1*^−/−^ fish after infection with 80 Mm - PDIM^−^. (J) Mean resident macrophage and monocyte recruitment from 5 to 150 mpi in the HBV of wild-type fish after infection with 80 live or heat-killed (HK) Mm - PDIM^−^. (K) Mean resident macrophage and monocyte recruitment from 5 to 120 mpi in the HBV of wild-type fish after infection with 80 wild-type or PDIM^−^ Mm. Results in (F) and (H) through (K) representative of at least three independent experiments.

For mycobacterial infection, our findings that resident macrophages are rapidly recruited through a PGL- and Ccl2-independent pathway followed by PGL- and Ccl2-dependent monocyte recruitment, led us to ask whether monocyte recruitment was dependent on resident macrophage recruitment. We first used zebrafish larvae depleted of myeloid cells (by morpholino-mediated inhibition of myeloid transcription factor *pu.1* expression; Clay et al., 2007) and evaluated *ccl2* expression following intravenous infection with PGL-competent Mm. Myeloid-deficient fish were unable to induce *ccl2* consistent with myeloid cells being responsible for Ccl2 production in response to mycobacterial infection (Figure 2A). Next, to specifically determine whether resident macrophages could induce *ccl2*, we infected bacteria into the HBV and used in situ hybridization analysis (Clay et al., 2007) with an antisense *ccl2* RNA probe. At 1 hr post infection, when the recruited phagocytes comprise almost entirely resident macrophages (Figure 1E), *ccl2*-positive phagocytes were present, but only following wild-type Mm infection and not PGL-deficient Mm infection (Figures 2B–2D). Together, these data showed that resident macrophages, like peripheral monocytes, induce *ccl2* in response to mycobacteria. This induction is PGL-dependent in both cases, suggesting the presence of a common activation program in both cell types.

Next, to directly test whether resident macrophages are required for monocyte recruitment, we used zebrafish mutants in which colonization of the brain by resident macrophages is delayed due to a genetic mutation in colony-stimulating factor receptor 1 (CSF1R) (Herbomel et al., 2001). Therefore, at the time of our recruitment assay (2 days post fertilization), *csf1r*^−/−^ fish have normal numbers of circulating monocytes but very few resident macrophages (Herbomel et al., 2001, Pagán et al., 2015) (Figure 2E). Following wild-type Mm infection into the HBV of *csf1r*^−/−^ fish, resident macrophage recruitment was decreased and delayed, consistent with the lack of available cells in the brain (Figure 2F). Importantly, monocyte recruitment was also markedly decreased, consistent with our hypothesis that resident macrophages mediate monocyte recruitment (Figure 2F). In conjunction with our earlier finding that mycobacterial PGL was also required for monocyte recruitment (Figure 1B), these findings supported a model where resident macrophages, recruited in response to generic bacterial signals, engulf the mycobacteria. Mycobacterial PGL then induces them to express Ccl2 that mediates monocyte recruitment. Because PGL is heat-stable (Onwueme et al., 2005), this model would predict that heat-killed PGL-expressing Mm would both induce Ccl2 and recruit monocytes. It did neither, suggesting that live PGL-expressing mycobacteria are required to recruit monocytes through Ccl2 induction in resident macrophages (Figures 2G and 2H). Notably, heat-killed bacteria did recruit resident macrophages (Figure 2H), consistent with the secreted factor responsible for resident macrophage recruitment being heat-stable.

Our finding that peripheral monocytes were dependent on signals from resident macrophages to participate in mycobacterial infection was surprising, and we wondered whether this requirement was unique to PGL-expressing mycobacteria. To test this, we used PDIM-deficient Mm, which recruits monocytes through TLR-Myd88 signaling, not Ccl2. *Csf1r*^−/−^ zebrafish recruited monocytes normally to PDIM-deficient bacteria (Figure 2I). Moreover, in contrast to wild-type mycobacteria, heat-killed PDIM-deficient Mm recruited monocytes (Figure 2J). These results suggested a passive detection of the surface-exposed TLR ligands of this mutant bacterium (Cambier et al., 2014b) in contrast to an active recruitment process mediated through live PGL-expressing bacteria. A head-to-head comparison of the recruitment kinetics of wild-type and PDIM-deficient strains revealed earlier monocyte recruitment to PDIM-deficient bacteria (Figure 2K), consistent with their recruitment to this strain being independent of resident macrophages. In sum, resident macrophages specifically promote Ccl2-dependent monocyte recruitment in response to virulent mycobacteria, and this is dependent on mycobacterial PGL.

Taken together, our findings suggest that heat-stable bacterial PAMPs of PDIM-deficient Mm trigger a program of microbicidal monocyte recruitment that is not dependent on resident macrophages. In contrast, when bacterial PAMPs are masked by PDIM, PGL-mediated recruitment of permissive monocytes is absolutely dependent on both resident macrophages and live bacteria, suggesting an active bacterial manipulation of these default first-responders.

### Mm PGL Recruits Monocytes through STING-Dependent CCL2 Induction

**Figure 3:**
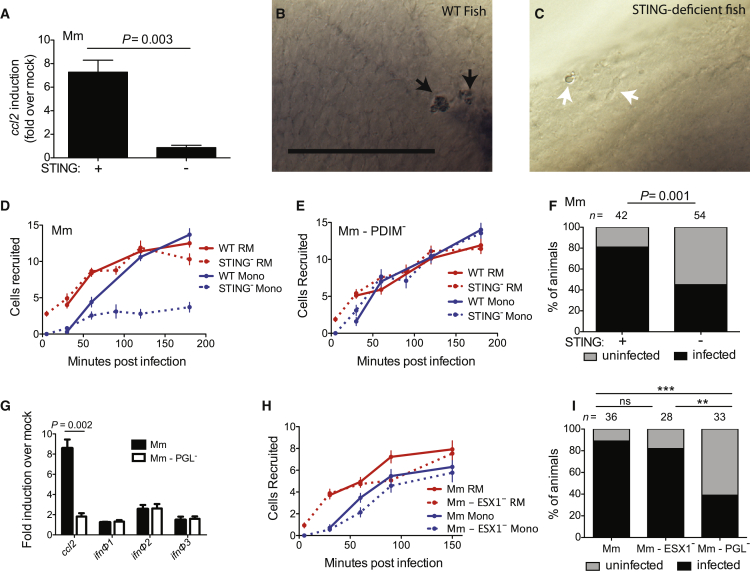
Mm PGL Recruits Monocytes through STING-Dependent *ccl2* Induction (A) *ccl2* messenger RNA levels (mean ± SEM of three biological replicates) induced at 3 hr after caudal vein infection of 2 dpf wild-type or Sting-deficient fish with 250–300 wild-type Mm. Student’s unpaired t test. (B and C) In situ hybridizations against zebrafish *ccl2* mRNA following hindbrain ventricle infections with 80 wild-type Mm into wild-type (B) or Sting-deficient (C) zebrafish. Black arrows, *ccl2* mRNA-positive phagocytes; white arrows *ccl2* mRNA-negative phagocytes. Scale bar, 50μm. Results representative of three independent experiments. (D) Mean resident macrophage and monocyte recruitment from 5 to 180 mpi in the HBV of wild-type or Sting-deficient fish after infection with 80 wild-type Mm. (E) Mean resident macrophage and monocyte recruitment from 5 to 180 mpi in the HBV of wild-type or Sting-deficient fish after infection with 80 Mm - PDIM^-^. (F) Percentage of infected (black) or uninfected (gray) wild-type or Sting-deficient fish 5 dpi with 1-3 wild-type Mm into the HBV. n = number of larvae per group. Results representative of two independent experiments. Significance testing done using Fisher’s exact test. (G) *ccl2*, *ifnΦ1*, *ifnΦ2*, and *ifnΦ3* mRNA levels (mean ± SEM of three biological replicates) induced at 3 hr after caudal vein infection of 2 dpf wild-type fish with 250–300 wild-type Mm. Significance testing done using Student’s unpaired t test for each gene. p = 0.002 for *ccl2*, all other comparisons not significant. (H) Mean resident macrophage and monocyte recruitment from 5 to 150 mpi in the HBV of wild-type fish after infection with 80 wild-type or ESX-1-deficient (ESX1^−^) Mm. (I) Percentage of infected (black) or uninfected (gray) wild-type fish 5 dpi of 1–3 wild-type, ESX1^−^, or PGL^−^ Mm into the HBV. n = number of larvae per group. Significance testing done using Fisher’s exact test for comparisons shown. ^∗∗^p < 0.01, ^∗∗∗^p < 0.001. Results representative of two independent experiments. Results in (D), (E), and (H) representative of three independent experiments.

How might PGL induce Ccl2 in resident macrophages? Because PGL operated in the context of live bacteria, we wondered whether a cytosolic sensing pathway was involved. Activation of the cytosolic signaling pathway STING can induce CCL2 (Chen et al., 2011), so we tested whether Sting was the intermediary in PGL-mediated Ccl2 induction. Sting depletion using a splice-blocking morpholino (Ge et al., 2015) resulted in a lack of *ccl2* induction in response to wild-type Mm in both peripheral monocytes (Figure 3A) and resident macrophages (Figures 3B and 3C). Consistent with the inability to induce *ccl2* in resident macrophages, Sting-deficient animals had reduced monocyte recruitment to Mm (Figure 3D). The initial recruitment of resident macrophages in these animals was intact, consistent with the prior finding that it was PGL-independent. Importantly, Sting-deficient animals recruited monocytes normally to PDIM-deficient Mm confirming that their inability to elicit monocytes was specifically in the context of Ccl2-mediated and not Myd88-dependent monocyte recruitment (Figure 3E). Finally, our model would predict that like Ccr2 deficiency, Sting deficiency should compromise the ability of wild-type bacteria to establish infection. Mycobacterial infectivity can be stringently tested by infecting animals with very low inocula that resemble human infection; in the zebrafish we have developed an infectivity assay which determines how many animals remain infected 4–5 days after infection with 1–3 mycobacteria (Cambier et al., 2014b). Using this infectivity assay, we found that wild-type Mm had reduced infectivity in Sting-deficient animals (Figure 3F), similar to PGL-deficient bacteria in wild-type animals and wild-type bacteria in Ccr2-deficient animals (Cambier et al., 2014b).

STING can induce CCL2 either through type I interferons (IFNs) (Cepok et al., 2009, Conrady et al., 2013), or independently of them (Chen et al., 2011). We evaluated expression of the zebrafish type I IFNs, *ifnΦ1-3*, that are induced during viral infection of larvae and adults, promote an antiviral gene program, and are protective against viral infection (Aggad et al., 2009). They were not induced appreciably at 3 hpi with wild-type Mm, and the minimal induction observed was not PGL-dependent (Figure 3G). As expected, *ccl2* was robustly induced in a PGL-dependent fashion (Figure 3G). This lack of dependence of type I IFNs on STING activation was distinct from the two previously reported pathways by which mycobacteria activate STING either through bacterial c-di-AMP or bacterial nucleic acid (Dey et al., 2015, Manzanillo et al., 2012). The latter of these requires the bacterial ESX-1 secretion system to permeabilize the bacterial phagosome in order to induce type I IFN (Siméone et al., 2015) that activates STING (Manzanillo et al., 2012). Having ruled out the involvement of type I IFNs, we used functional studies to further rule out that STING activation of our pathway was ESX-1-dependent. If STING activation of CCL2 is reliant on ESX-1 induction of type I IFNs, then monocyte recruitment should be ESX-1-dependent. We found that it was not. ESX-1 mutant bacteria recruited both resident macrophages and monocytes normally to the initially infecting bacteria (Figure 3I). Consistent with this finding, ESX-1-deficient Mm established infection at wild-type levels (Figure 3J). Our prior work has found that ESX-1 partners with host MMP9 to accelerate macrophage recruitment to the forming granuloma (Volkman et al., 2004). These new findings showed that initial macrophage recruitment occurs through a distinct mechanism—PGL-dependent activation of STING that directly induces CCL2. It is not surprising that this process is ESX-1 independent because of the timing of *ccl2* induction (prior to 3 hr post infection) versus ESX-1-induced phagosome permeabilization which takes ∼24 hr (Siméone et al., 2015). Whether PGL is directly sensed by STING or works through an intermediary remains to be determined. It also remains to be determined how PGL or its intermediary contacts the cytosolic signaling pathway. One possibility is through mycobacterial vesicles that can be secreted out of the phagosomes of infected macrophages (Rhoades et al., 2003). Formation of these vesicles requires bacterial viability (Athman et al., 2015) but not ESX-1 (Bhatnagar and Schorey, 2007), both consistent with our findings.

### PGL-Expressing Bacteria Can Transfer from Resident Macrophages to Monocytes

**Figure 4:**
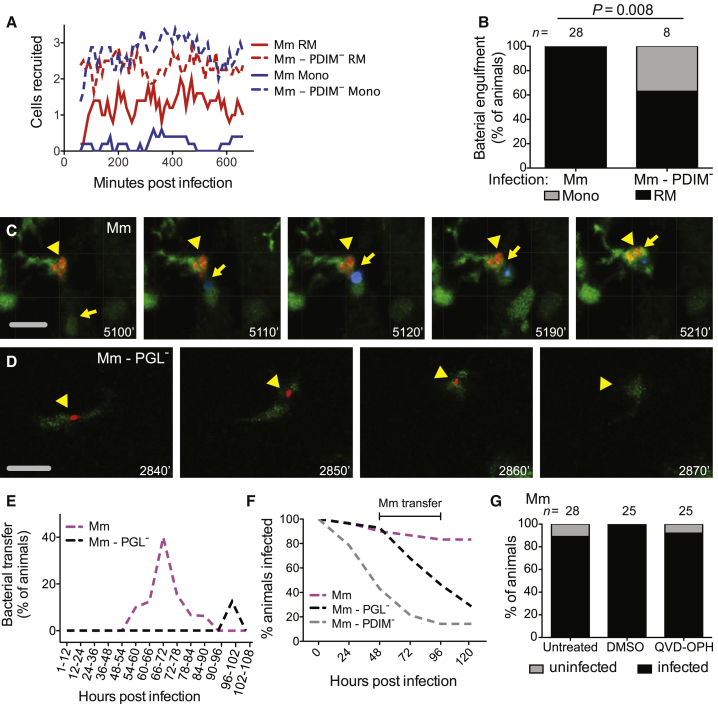
PGL Promotes Intercellular Bacterial Transfer and Prevents Bacterial Clearance (A) Mean (≥5 biological replicates) number of resident macrophages and monocytes occupying the HBV at each time point, quantified every 10 min from 1 to 11 hpi in Tg (*mpeg1::yfp*) fish with green fluorescent macrophages after infection with 1–3 wild-type or PDIM^−^ red fluorescent Mm. (B) Percentage of fish where the infecting bacteria were phagocytosed by a resident macrophage (black) or a monocyte (gray) over the first 11 hr following infection of Tg (*mpeg1*:YFP) fish in the HBV with red fluorescent 1–3 wild-type or PDIM^−^ Mm. n = number of larvae per group. Significance testing done using Fisher’s exact test. Results representative of three independent experiments. (C) Representative images from a time-lapse movie of a bacterial transfer event. Uninfected Hoechst positive (blue fluorescence) monocyte (yellow arrow) is seen phagocytosing an infected cell (yellow arrowhead). Scale bar, 50 μm. Time stamp, mpi. (D) Representative images from a time-lapse movie showing an infected macrophage (green fluorescent) clearing red fluorescent PGL^−^ Mm (yellow arrowhead). Scale bar, 50μm. Time stamp, mpi. (See also Movies S1 and S2 and Tables S1 and S2.) (E) Quantification of bacterial transfer events from experiments represented by (C) and (D). Percentage of animals demonstrating a transfer event during the designated imaging time block. (F) Percentage of animals remaining infected over the first 5 days of infection with 1–3 wild-type, PGL^−^, or PDIM^−^ Mm into the HBV of wild-type fish. Numbers of fish infected with each Mm strain: 30 wild-type, 28 PGL^−^, and 28 PDIM^−^. Results representative of two separate experiments. (G) Percentage of infected (black) or uninfected (gray) untreated, DMSO control, or QVD-OPH treated wild-type fish 5 dpi with 1-3 wild-type Mm into the HBV. n = number of larvae per group. Results representative of two separate experiments.

Human TB is thought to result from infection with only 1–3 bacteria (Bates et al., 1965, Cambier et al., 2014a, Wells et al., 1948). In the zebrafish, 1–3 Mm are sufficient to establish infection in the majority of zebrafish larvae provided that bacterial PGL and host Sting and Ccl2-Ccr2 are present; without these factors, infectivity is reduced (Figure 3F) (Cambier et al., 2014b). Therefore, it was important to examine myeloid cell recruitment in response to these low inocula where the role of PGL and CCR2 is most relevant. To enable a detailed temporal analysis of the HBV by time-lapse confocal microscopy, we used *mpeg::yfp* or *mpeg::tdtomato* transgenic zebrafish with fluorescent myeloid cells, again using Hoechst dye to distinguish monocytes from resident macrophages. Imaging each animal every 10 min from 1–11 hr post infection, we quantified the total number of resident macrophages and monocytes occupying the HBV at each time point. We observed that resident macrophages arrived early whereas monocytes were rarely seen during this period (Figure 4A and Table S1). In contrast, even with these low inocula, both cell types were recruited early to PDIM-deficient mutants (Figure 4A). Accordingly, when we analyzed the phagocytosis event for each bacterium, we found that wild-type bacteria were phagocytosed only by resident macrophages whereas PDIM-deficient bacteria were phagocytosed by both resident macrophages and monocytes (Figure 4B).

Previously, we had shown that the increased infectivity of PGL-competent bacteria is abrogated by Ccr2 deficiency (Cambier et al., 2014b). Now we had found that both PGL-competent and PGL-deficient bacteria are initially in resident macrophages that are recruited in a Ccr2-independent manner, with the critical difference between the two strains being whether there is subsequent recruitment of Ccr2-dependent monocytes or not. Taken together, the two findings suggested that these monocytes were responsible for the increased infectivity of PGL-competent bacteria. This could be because the monocytes comprised a more permissive niche into which the bacteria were transferring, or because their presence was modulating the microbicidal capacity of the originally-infected resident macrophages.

In order to determine whether bacteria were being transferred to new cells, we had to image infection for the first several days. Continuous imaging of the infection site in the same animal for several days is precluded by photobleaching. So we devised a strategy where we divided the infected larvae into 14 groups, and imaged each group for one of consecutive 6 or 12 hr periods that together spanned 4.5 days of infection (Table S2). For wild-type bacteria, transfer events were observed starting at 54 hr and peaking in the 66–72 hr window (Figure 4C and 4E and Movie S1). These transfers were accomplished as follows (Movie S1): the infected resident macrophage was approached by an uninfected peripheral monocyte. The cells then converged for a period of time before separating again, with the bacteria now being associated with the peripheral monocyte. Transfer events were not observed for PGL-deficient infection in the 66–72 hr window. (Figure 4E and Table S2). Thus, PGL-deficient bacteria largely remained within resident macrophages longer than wild-type bacteria. Furthermore, we documented clearance events of PGL-deficient Mm by the initially infected macrophage (Figure 4D and Movie S2). In contrast, clearance events were not observed during wild-type Mm infection.

To rigorously examine the kinetics of clearance in relation to the bacterial transfer events we had observed, we monitored ∼30 animals for bacterial clearance by imaging them once every 24 hr. Because wild-type bacteria only transfer into permissive monocytes starting at 54 hr, the differential clearance of wild-type and PGL-deficient bacteria should become apparent only after this time-point. This was the case (Figure 4F). In contrast, PDIM-deficient bacteria started to be cleared within 24 hr (Figure 4F) consistent with their recruiting microbicidal monocytes within 2 hr and being phagocytosed by them within 12 hr (Figures 4A and 4B).

Imaging of these early mycobacterium-beneficial transfer events revealed they were distinct in their cellular morphology from subsequent intercellular bacterial transfer observed in the forming granuloma, which is dependent on the apoptotic death of the infected macrophage, the bacterial contents of which are engulfed by newly arriving macrophages (Davis and Ramakrishnan, 2009). In contrast the PGL-dependent transfer event was characterized by movement of the “donor” infected resident macrophage until the time that it converged with the “recipient” peripheral monocyte (Movie S1). Because the ESX-1 locus promotes apoptosis of infected macrophages (Davis and Ramakrishnan, 2009), our finding that ESX-1-deficient Mm were not compromised during early infectivity (Figure 3H), suggested that efferocytosis is not mediating this transfer event. To confirm this, we used the pan-caspase inhibitor QVD-OPH that reduces apoptotic cells substantially (7.2-fold) in the context of Mm infection of the zebrafish (Yang et al., 2012). QVD-OPH treatment did not reduce the early infectivity of Mm (Figure 4G), further suggesting that this transfer event is not dependent on efferocytosis. Rather, transfer was occurring between living cells, similar to the findings that, following intimate contact between macrophages in culture, intracellular Gram-negative pathogens can transfer between the two cells in a process known as trogocytosis (Steele et al., 2016).

Together, these findings are consistent with the model that PGL-competent bacteria transfer into the permissive monocytes they recruit. Conversely, our finding that PGL-deficient mycobacteria have a more prolonged sojourn in resident macrophages in which they are cleared, suggests that resident macrophages are more microbicidal than Ccl2-recruited monocytes.

### Resident Macrophages Are More Microbicidal than Monocytes

**Figure 5:**
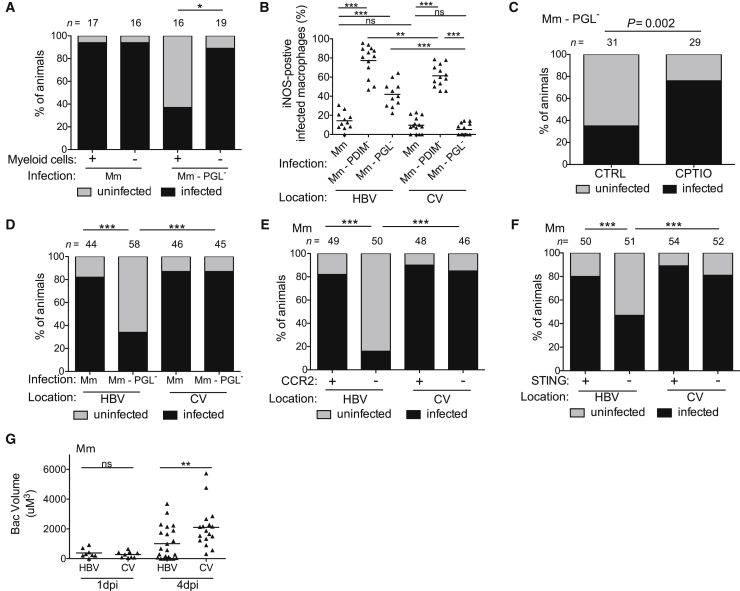
Resident Macrophages Are More Microbicidal than Monocytes (A) Percentage of infected (black) or uninfected (gray) wild-type or myeloid-deficient fish at 5 dpi after HBV infection with 1–3 wild-type or PGL^−^ Mm. n = number of larvae per group. (B) Percentage of iNOS-positive infected myeloid cells in the HBV or CV at 3 dpi with 80 wild-type, PDIM^−^ or PGL^−^ Mm. (C) Percentage of infected (black) or uninfected (gray) wild-type fish at 5 dpi after HBV infection with 1–3 PGL^−^ Mm. Control, CTRL; Reactive nitrogen species scavenger CPTIO. n = number of larvae per group. (D) Percentage of infected (black) or uninfected (gray) wild-type fish at 5 dpi with 1–3 wild-type or PGL^−^ Mm into the HBV or CV. n = number of larvae per group. (E) Percentage of infected (black) or uninfected (gray) wild-type or Ccr2-deficient fish at 5 dpi with 1–3 wild-type Mm in the HBV or CV. n = number of larvae per group. (F) Percentage of infected (black) or uninfected (gray) wild-type or Sting-deficient fish at 5 dpi with 1–3 wild-type Mm in the HBV or CV. n = number of larvae per group. (G) Mean bacterial volume at 1 and 4 dpi with a single wild-type Mm bacterium in the HBV or CV of wild-type fish. Results in (A)–(G) representative of three independent experiments. (B) and (G) significance testing done using one-way ANOVA, with Bonferroni’s post-test for comparisons shown. (A) and (C)–(F) significance testing done using Fisher’s exact test for the comparisons shown. ^∗^p < 0.05, ^∗∗^p < 0.01, ^∗∗∗^p < 0.001.

Our findings linking increased time in the resident macrophage to increased bacterial killing suggested that resident macrophages are more microbicidal than Ccr2-recruited monocytes. To address this question, we took advantage of our finding that following infection with 1–3 bacteria, only resident macrophages harbor PGL-deficient bacteria for at least the first 4.5 days (Figure 4E). We found that in Pu.1 morphant animals lacking myeloid cells and therefore the resident macrophage niche they occupied at this stage, PGL-deficient bacteria were able to establish infection at wild-type levels (Figure 5A). These data further suggested that resident macrophages are microbicidal to PGL-deficient bacteria. We found that PGL-deficient infection resulted in more inducible nitric oxide synthase (iNOS)-positive cells than wild-type infection (Figure 5B). This was similar to the case of the PDIM-deficient mutant whose TLR-recruited monocytes express more iNOS than Ccl2-elicited monocytes (Figure 5B) (Cambier et al., 2014b). However, since PGL-deficient Mm recruits only resident macrophages, the increased iNOS production must be coming from the resident macrophages—i.e., resident macrophages, like TLR-recruited monocytes, also produce more iNOS than Ccl2-elicited permissive monocytes following infection. If this were the case then following delivery directly to monocytes via caudal vein infection (Figure 1A), PGL-deficient bacteria should result in the same low number of infected iNOS-positive cells as wild-type bacteria, and they did (Figure 5B). PDIM-deficient infection induced more iNOS in the caudal vein also (Figure 5B), suggesting that myeloid cells responding to PDIM-deficient bacteria are more activated regardless of location. Finally, we showed that the increased iNOS expression in the resident macrophages contributed to their increased microbicidal activity, as it does for TLR-recruited monocytes (Cambier et al., 2014b)—treatment of animals with the nitric oxide scavenger CPTIO increased the infectivity of PGL-deficient bacteria delivered into the HBV (Figure 5C). Together these results suggested that the reduced infectivity of PGL-deficient bacteria is due to their prolonged sojourn in resident macrophages. If so, then the infectivity of PGL-deficient bacteria should be restored when delivered directly to monocytes by intravenous infection. It was (Figure 5D), and this result further showed that mycobacterial PGL does not protect mycobacteria from the microbicidal activity of resident macrophages but rather promotes their escape into the more permissive monocytes. Both Ccr2-deficiency and Sting-deficiency, which produced the expected decrease in infectivity of wild-type Mm upon hindbrain ventricle infection, failed to do so when the bacteria were delivered directly to monocytes through caudal vein infection (Figures 5E and 5F). Together, these findings highlighted the role of STING and CCL2 as early host susceptibility factors that work by enabling recruitment of peripheral monocytes to sites of infection.

Finally, we asked whether the 54–90 hr sojourn in resident macrophages was at all detrimental to wild-type bacteria. The infectivity assay we had used so far only assessed whether the animals had cleared the bacteria or not, and not the extent of bacterial growth in the animals that did not clear them. We now tested this following infection of animals with a single bacterium. We found twice as much bacterial growth in the caudal vein compared to the HBV (Figure 5G). Together these results show that resident macrophages are more microbicidal than the permissive monocytes to which the wild-type bacteria eventually gain access. Moreover, the resident macrophage plays a growth-restrictive role even to wild-type PGL-expressing bacteria during the truncated time period that they remain in it.

### Human Alveolar Macrophages Rapidly Secrete CCL2 after Mycobacterial Infection in a PGL-Dependent Fashion

Our prior work had shown that pathogenic mycobacteria establish infection by recruiting and infecting permissive monocytes while having specialized strategies to avoid recruiting microbicidal cells, neutrophils (Yang et al., 2012), and TLR-stimulated monocytes (Cambier et al., 2014b). The latter strategy requires that mycobacteria initiate infection in the lower lung, so as to avoid the TLR-stimulated microbicidal monocytes by the mucosal flora of the upper airway. The present work had now identified the resident macrophage as another default rapid first-responder microbicidal cell that mycobacteria cannot avoid even in the lower airways. It must therefore co-opt them into their escape strategy by inducing them to secrete CCL2. In terms of human relevance of our zebrafish findings, our findings that resident macrophages are more microbicidal than peripheral monocytes already had support from human studies: human alveolar macrophages have substantial mycobactericidal activity ex vivo, in contrast to peripheral blood monocytes which not only fail to kill mycobacteria but are growth-permissive (Aston et al., 1998, Hirsch et al., 1994, Rich et al., 1997, van Zyl-Smit et al., 2014). Moreover, consistent with our findings, the microbicidal activity of human alveolar macrophages is at least in part mediated by nitric oxide (Hirsch et al., 1994).

**Figure 6:**
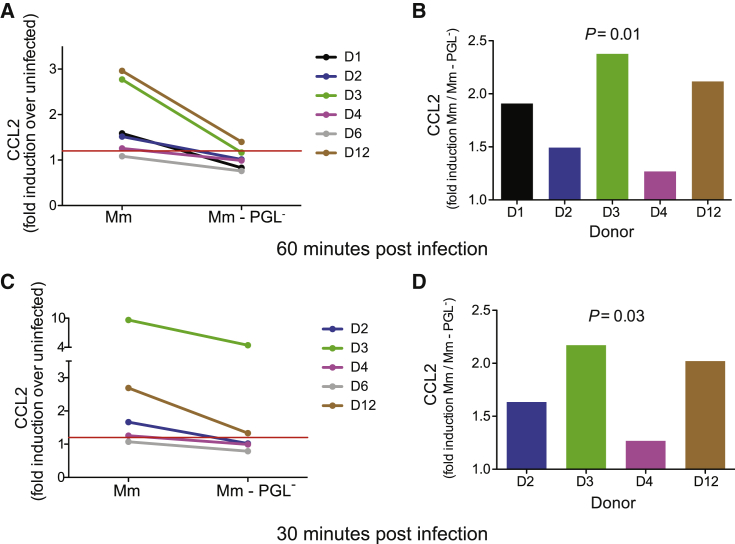
PGL-Dependent CCL2 Protein Production following Mm Infection of Human Alveolar Macrophages (A and C) Fold increase (over uninfected cells) in CCL2 protein levels in the supernatant of primary human alveolar macrophages following a 60 min (A) or 30 min (C) infection with wild-type Mm or PGL-deficient Mm. (B and D) The same data as in (A) and (C) analyzed as fold increase in CCL2 of wild-type Mm over PGL- Mm at 60 min (B) and 30 min (D) post infection. Significance testing done using a one sample t test to a hypothetical value of 1, corresponding to the null hypothesis that PGL does not influence CCL2 production following infection. (See also Table S3.)

Our model would further predict that human alveolar macrophages would rapidly produce CCL2 upon mycobacterial infection in a PGL-dependent fashion. To test this prediction, we performed a pilot experiment with human alveolar macrophages obtained by bronchoalveolar lavage. We infected them with either PGL-expressing or PGL-deficient Mm. CCL2 was induced in a PGL-dependent fashion at 60 min post-infection (Figure 6A and 6B, Donor 1). We then recruited 12 additional donors and infected their alveolar macrophages with PGL-expressing or PGL-deficient mycobacteria as well as with LPS (100 ng/ml), a known CCL2 inducer. LPS induced CCL2 (> 1.2 fold over uninfected) in 5 of 12 donors suggesting that the remaining were not capable of inducing CCL2 rapidly in response to a known inducer (Table S3). The LPS-nonresponding macrophages also did not induce CCL2 upon mycobacterial infection (Table S3). This nonresponsiveness is consistent with significant donor variation in human alveolar macrophage cytokine secretion after mycobacterial infection (Keane et al., 2000). Of the LPS-responding macrophages, four of five induced CCL2 upon mycobacterial infection and this response was PGL-dependent (Figures 6A and 6B, and Table S3). In order to see whether CCL2 induction occurred even earlier than 60 min, we had collected supernatants at 30 min. Only those donor alveolar macrophages that induced CCL2 in response to LPS and mycobacterial infection at the 60 min time point, did so at the 30 min time point (Figure 6C and Table S3). Again, CCL2 induction was PGL-dependent (Figures 6C and 6D). These experiments suggest that the rapid induction of CCL2 in human alveolar macrophages in response to mycobacterial infection is PGL-dependent.

## Discussion

By tracking the dynamics and kinetics of the earliest myeloid cell responses in the first hours of mycobacterial infection, we found that tissue-resident macrophages are the first cells to come in contact with any infecting bacteria in response to a ubiquitous heat-stable secreted bacterial signal. Arriving to virulent mycobacteria, resident macrophages were rapidly infected and could subsequently eradicate infection. In turn, mycobacterium’s counterstrategy to circumvent this first-line host defense that it cannot evade was to engineer its escape from these cells.

The PGL-STING-mediated pathway of *ccl2* induction was shared by both resident macrophages and monocytes. It is interesting then that mycobacteria deploy PGL-CCL2-mediated recruitment only initially. The involvement of CCL2 in subsequent monocyte recruitment to the forming granuloma is less clear. CCR2-deficient animals are not compromised for granuloma formation (C.J.C. and L.R., unpublished data). Rather monocyte recruitment to the granulomas is dependent on another bacterial virulence determinant, the ESX-1 locus that induces monocyte recruitment through induction of the host matrix metalloproteinase 9 (Volkman et al., 2010). Intercellular bacterial transfer in the granuloma requires the apoptotic death of a highly-infected macrophage that is then engulfed by multiple new recruits so as to expand the bacterial niche (Davis and Ramakrishnan, 2009). Therefore, this mechanism of granuloma expansion depends upon bacteria being in a growth-permissive cell and might be less effective when the bacteria are still within the more microbicidal-resident macrophage. In contrast, this work shows that PGL-induced CCL2 occurred even under the bacteriostatic or bactericidal conditions imposed by the resident macrophage, allowing even the few remaining bacteria to escape into permissive cells. On the other hand, it remains unclear why the PGL-CCL2 pathway becomes less relevant during granuloma formation. Perhaps the kinetics of ESX-1-MMP9 recruitment are faster, benefitting the bacterium by allowing for its greater intercellular expansion and spread at this stage.

We have recently shown that *Mycobacterium leprae*’s PGL-1, differing from Mm’s and Mtb’s PGL in the carbohydrate domain, is required for monocyte-mediated demyelination at a later step of the infection (Madigan et al., 2017). However, *M. leprae* also mediates recruitment of monocytes through CCL2-CCR2 signaling, suggesting that its specialized PGL-1 still retains the basal function of eliciting permissive monocytes to promote its infectivity at the first steps of infection. It is noteworthy that both PGL-mediated functions—establishment of infection and demyelination—are through manipulation of host myeloid cells (Madigan et al., 2017, and this work).

Our findings highlight not only both phylogenetic and ontogenic conservation of resident macrophage function but also suggest that different tissue resident macrophages—even the most specialized brain-resident macrophages (Casano and Peri, 2015)—all retain their primal function as sentries against invading pathogens (Epelman et al., 2014, Gordon et al., 2014). The finding that resident macrophages can make short shrift of mycobacteria, notoriously pernicious pathogens, is particularly noteworthy given their key role in tissue homeostasis (Epelman et al., 2014). It is curious that CCL2-elicited monocytes provided a safe-haven to mycobacteria as CCR2^+^ monocytes are broadly microbicidal against bacterial, fungal protozoan, and viral pathogens (Serbina et al., 2008). Indeed, these cells, also called inflammatory monocytes, are implicated in the pathogenesis of multiple inflammatory diseases affecting the brain, gut, and vascular system (Lauvau et al., 2014, Shi and Pamer, 2011). On the other hand, CCR2^+^ myeloid cells have been implicated in promoting an immunosuppressive tumor environment (Lesokhin et al., 2012). Our data identified a permissive role for these cells in the context of an important intracellular infection. Consistent with our findings, CCL2-recruited monocytes have been previously shown to be more permissive to Mtb growth in the lungs of mice (Antonelli et al., 2010), and mice overexpressing CCL2 were found to be more susceptible to challenge with Mtb (Rutledge et al., 1995). Their reduced microbicidal capacity in response to mycobacterial infection might simply reflect the masking of activating TLR ligands by mycobacteria, though it is notable that even in the absence of TLR-mediated activation, resident macrophages are more microbicidal to mycobacteria than monocytes. Of course TB is a complex infection and it is possible that as infection progresses, these same inflammatory monocytes could take on a host-beneficial role in delivering mycobacterial antigens to pulmonary lymph nodes to eventually lead to antigen-specific T cell responses (Samstein et al., 2013). However, even this role might have complex consequences—while T cell responses are clearly protective for individuals, they might also be paradoxically benefitting bacteria by promoting transmission to new individuals (Comas et al., 2010). Overall, our findings add to the discussion of the plasticity and context-dependent function of myeloid cells, for which there is increasing appreciation particularly with the advent of in vivo studies suggesting that myeloid cell functions defy rigid classifications (Martinez and Gordon, 2014, Murray et al., 2014).

Finally, we note that while evolutionary ancestors of Mtb e.g., Mm and *Mycobacterium cannetti* uniformly express PGL, the prevalence of PGL-expression in modern-day Mtb strains is not clear (Gagneux et al., 2006, Pang et al., 2012). This work emphasizes the need to assess the prevalence of PGL-positive strains, and to thoroughly examine TB transmission epidemiology in regions where PGL-expressing strains abound, while devising therapeutic strategies to block PGL to prevent TB infection and transmission.

## STAR★Methods

### Key Resources Table

**Table:** 

REAGENT or RESOURCE	SOURCE	IDENTIFIER
**Antibodies**		

Anti-iNOS	BD Biosciences	Cat#610333
Anti-DIG-AP	Sigma	Cat# 11093274910

**Bacterial and Virus Strains**		

*M. marinum* M strain transformed with pMSP12:tdTomato or pMSP12:wasabi	(Takaki et al., 2013)	derivatives of ATCC #BAA-535
*Δpks15 M. marinum* M strain transformed with pMSP12:tdTomato or pMSP12:wasabi	(Cambier et al., 2014b)	N/A
Δ*mmpL7 M. marinum* M strain transformed with pMSP12:tdTomato or pMSP12:wasabi	(Cambier et al., 2014b)	N/A
*Δesx1 M. marinum* M strain transformed with pMSP12:tdTomato or pMSP12:wasabi	(Volkman et al., 2004)	N/A
*S. aureus* Newman strain expressing pOS1-SdrC-mCherry #391	J. Bubeck Wardenburg	N/A
*P. aeruginosa* PAO1 expressing GFP	(Brannon et al., 2009)	N/A

**Chemicals, Peptides, and Recombinant Proteins**		

cPTIO (carboxy-α-phenyltetramethylnitronyl nitroxide)	Sigma	CAS # 148819-94-7
Hoechst 33342	Thermo Fisher	CAS # 23491-52-3
QVD-OPH ((3S)-5-(2,6-Difluorophenoxy)-3-[[(2S)-3-methyl-1-oxo-2-[(2-quinolinylcarbonyl)amino]butyl]amino]-4-oxo-pentanoic acid hydrate)	Sigma	CAS# 1135695-98-5

**Experimental Models: Organisms/Strains**		

Zebrafish: wildtype AB	University of Washington	ZFIN ID: ZDB-GENO-960809-7
Zebrafish: Tg(*mpeg1:Brainbow*)^w201^	(Pagán et al., 2015)	ZFIN ID: ZDB-FISH-151204-7
Zebrafish: Tg(*lysC:EGFP*)^nz117^	(Hall et al., 2007)	ZFIN ID: ZDB-FISH-150901-28454
Zebrafish: Tg(*mpeg1:YFP*)^w200Tg^	(Roca and Ramakrishnan, 2013)	ZFIN ID: ZDB-FISH-150901-6828
Zebrafish: *csf1ra*^*j4blue*^ (csf1r mutants)	(Parichy et al., 2000)	ZFIN ID: ZDB-FISH-150901-1291

**Oligonucleotides**		

*ccl2* mRNA forward primer for qPCR, sequence: GTCTGGTGCTCTTCGCTTTC	(Cambier et al., 2014b)	N/A
ccl2 mRNA reverse primer for qPCR, sequence: TGCAGAGAAGATGCGTCGTA	(Cambier et al., 2014b)	N/A
beta actin mRNA forward primer for qPCR, sequence: AGAGGGAAATCGTGCGTGAC	(Ramirez-Carrozzi et al., 2009)	N/A
beta actin mRNA reverse primer for qPCR, sequence: CAATAGTGATGACCTGGCCGT	(Ramirez-Carrozzi et al., 2009)	N/A
*ifnΦ1* mRNA forward primer for qPCR, sequence: TTAATACACGCAAAGATGAGAACTC	this paper	N/A
*ifnΦ1* mRNA reverse primer for qPCR, sequence: GCCAAGCCATTCGCAAGTAG	this paper	N/A
*ifnΦ2* mRNA forward primer for qPCR, sequence: CCTCTTTGCCAACGACAGTT	this paper	N/A
*ifnΦ2* mRNA reverse primer for qPCR, sequence: CGGTTCCTTGAGCTCTCATC	this paper	N/A
*ifnΦ3* mRNA forward primer for qPCR, sequence: GAGGATCAGGTTACTGGTGT	this paper	N/A
*ifnΦ3* mRNA reverse primer for qPCR, sequence: GTTCATGATGCATGTGCTGTA	this paper	N/A
*tmem173* (STING) forward primer for morpholino efficiency screen, sequence: CTGCTGGACTGGGTTTTCTTACTC3	this paper	N/A
*tmem173* (STING) reverse primer for morpholino efficiency screen, sequence: TGGGTGATCTTGTAGACGCTGTTA	this paper	N/A
*pu.1* morpholino component 1, sequence: CCTCCATTCTGTACGGATGCAGCAT	(Clay et al., 2007)	N/A
*pu.1* morpholino component 2, sequence: GGTCTTTCTCCTTACCATGCTCTCC	(Clay et al., 2007)	N/A
*ccr2* morpholino, sequence: AACTACTGTTTTGTGTCGCCGAC	(Cambier et al., 2014b)	N/A
*myD88* morpholino, sequence: GTTAAACACTGACCCTGTGGATCAT	(Bates et al., 2007)	N/A
*tmem173* (STING) morpholino sequence: TGGAATGGGATCAATCTTACCAGCA	this paper	N/A
*ccl2* mRNA forward primer for design of in situ probe sequence: GTCAGCTAGGATCCATGAGGCCGTCCTGCATCC	this paper	N/A
*ccl2* mRNA reverse primer for design of in situ probe sequence: GTCAGCTATCTAGATTAGGCGCTGTCACCAGAG	this paper	N/A

**Recombinant DNA**		

pCS2+ plasmid	Marc Kirschner	Addgene #17095

**Critical Commercial Assays**		

Human MCP-1 (CCL2) chemokine kit	Meso Scale Discovery	Cat.# K151AYA

**Software and Algorithms**		

Imaris	Bitplane	N/A
Prism	GraphPad	N/A

### Contact for Reagent and Resource Sharing

*Further information and requests for resources and reagents should be directed to and will be fulfilled by the Lead Contact, Lalita Ramakrishnan* (lr404@cam.ac.uk).

### Experimental Models and Subject Details

#### Zebrafish Husbandry and Infections

Wild-type AB (University of Washington), *csf1ra*^*j4blue*^ homozygous mutant (*csf1r*^*−/−*^) zebrafish (Parichy et al., 2000), Tg(*mpeg1:YFP)*^*w200*^ (Roca and Ramakrishnan, 2013), and Tg(*mpeg1:Brainbow)*^*w201*^ (expressing tdTomato) (Pagán et al., 2015), and the Tg(*lyz:EFGP*)^nz117^ (Hall et al., 2007) lines were maintained in buffered reverse osmotic water systems. Fish were fed twice daily a combination of dry feed and brine shrimp and were exposed to a 14 hr light, 10 hr dark cycle to maintain proper circadian conditions. Larvae (of undetermined sex given the early developmental stages used) were infected at 48 hr post-fertilization (hpf) via caudal vein (CV) or hindbrain ventricle (HBV) injection using single-cell suspensions of known titer (Takaki et al., 2012, Takaki et al., 2013). Number of animals to be used for each experiment was guided by pilot experiments or by past results with other bacterial mutants and/or zebrafish. On average 35 to 40 larvae per experimental condition were required to reach statistical significance and each experiment was repeated at least twice. Larvae were randomly allotted to the different experimental conditions. All experiments where *csf1r*^*−/−*^ zebrafish were used, *csf1r*^*−/−*^ were either in-crossed or outcrossed to wild-type ABs to generate *csf1r*^*+/−*^ which are phenotypically wild-type (Pagán et al., 2015). The zebrafish husbandry briefly described above and all experiments performed on them were in compliance with guidelines from the UK Home Office (Cambridge experiments) and in compliance with the U.S. National Institutes of Health guidelines and approved by the University of Washington Institutional Animal Care and Use Committee (Seattle experiments) and the Stanford Institutional Animal Care and Use Committee (Stanford experiments).

#### Human Alveolar Macrophage Collection

Human alveolar macrophages (AMs) were retrieved at bronchoscopy as approved by the Research Ethics Committee of St. James’s Hospital (Reference number 2008/17/17), and previously reported (Berg et al., 2016, O’Leary et al., 2014). Briefly all donors were patients undergoing clinically indicated bronchoscopy and written informed consent for retrieving additional bronchial washings for research was obtained prior to the procedure. Thirteen donors were recruited to this study, of which 8 were male and 5 were female. The mean age of donors was 56yrs ± 3.4yr, with a range 32-70yrs. Bronchial washing fluid was filtered through a 100 μm nylon strainer (BD Falcon, BD Bioscience, Belgium) and centrifuged at 390 g for 10min. Alveolar macrophages were resuspended in RPMI 1640 culture media supplemented with 10% fetal bovine serum (FBS, GIBCO), 2.5ug/ml fungizone and 50 μg/ml cefotaxime. AMs were seeded at a density of 5 × 10^4^ cells/well in 96-well plates (Corning Costar, Nijmegen, Netherlands). AMs were purified by plastic adherence, non-adherent cells were removed by washing after 24hrs.

### Method Details

#### Bacterial Strains and Methods

Mm strain M (ATCC BAA-535) Δ*mmpL7,* Δ*pks15,* and *Δesx-1* mutants expressing either TdTomato or Wasabi under the control of the *msp12* promoter (Cambier et al., 2014b, Takaki et al., 2013) were grown under hygromycin (Mediatech) selection in 7H9 Middlebrook’s medium (Difco) supplemented with oleic acid, albumin, dextrose, and Tween-80 (Sigma). To prepare heat-killed Mm, bacteria were incubated at 80°C for 20 min. To prepare bacterial supernatants, bacteria were grown to an OD600 of 0.6, pelleted and the supernatant was then filtered twice through a 0.2μm filter. The *P. aeruginosa* PAO1 fluorescent strain has been described (Brannon et al., 2009). The *S. aureus* Newman strain expressing pOS1-SdrC-mCherry #391 was a gift from Dr. Juliane Bubeck Wardenburg.

#### Bead Injections

Sterile red-fluorescent 1μm beads (Thermo-Fisher Scientific F8821) were diluted ten fold with sterile PBS resulting in 3.64 × 10^3^ beads/nL. Approximately 5 nL of the bead mixture was injected into the hindbrain ventricle of 2 dpf larvae for a total of 1.8 x10^4^ beads per larva.

#### iNOS Immunofluorescence

To detect iNOS in infected larvae, larvae were euthanized by tricaine overdose, fixed overnight at 4°C in 4% paraformaldehyde (Sigma), permeabilized for 30 min with proteinase K (Thermofisher) at 10μg/mL in PBST (PBS + 0.1% Tween20 (Sigma)), then stained overnight at 4°C in iNOS antibody (see Key Resources Table) diluted 1:200, as described (Cambier et al., 2014b). After washing in PBST, secondary antibodies conjugated to Alexa Fluors (Molecular Probes) were added at 1:500 and incubated overnight at 4°C.

#### QVD-OPH and CPTIO Treatment

CPTIO or QVD-OPH (Sigma) was used at a final concentration of 50 mM and 50 μM, respectively, in 0.5% dimethylsulphoxide in fish water. Control fish were incubated in 0.5% dimethylsulphoxide only. Fish were incubated immediately following infection and fresh inhibitor was added every 24 hr until experiment end point.

#### Confocal Microscopy and Image-Based Quantification of Infection

Larvae were embedded in 1.5% agarose (low melting point) (Davis and Ramakrishnan, 2009). A series of z stack images with a 2 μm step size was generated through the infected HBV, using the galvo scanner (laser scanner) of the Nikon A1 confocal microscope with a 20x Plan Apo 0.75 NA objective. Bacterial burdens were determined by using the 3D surface-rendering feature of Imaris (Bitplane Scientific Software) (Yang et al., 2012).

#### Hindbrain Kinetic Assays

Macrophage recruitment assays were performed as previously described (Takaki et al., 2012), 2 dpf zebrafish were injected in the HBV with the bacterial strain or reagent and dose reported in the figure legends. At the specified time post injection, the number of myeloid cells in the HBV was quantified using differential interference contrast microscopy as described below. For assays distinguishing resident macrophages from monocytes, 200 μg/ml Hoechst 33342 (ThermoFisher) was injected via the caudal vein as previously described (Davis and Ramakrishnan, 2009) 2 hr prior to infection into the HBV. Differential interference contrast and fluorescent imaging using Nikon’s Eclipse E600 was done every ∼30 min to identify resident macrophages (Hoechst negative) and monocytes (Hoechst positive). Objectives used in this assay included 20x Plan Fluor 0.5 NA and 40x Plan Fluor 0.75 NA.

#### Morpholinos

The Sting morpholino 5′TGGAATGGGATCAATCTTACCAGCA3′ (see Key Resources Table) was designed to block the exon 2 intron 2 border. The following primer pair 5′CTGCTGGACTGGGTTTTCTTACTC3′ and 5′TGGGTGATCTTGTAGACGCTGTTA3′ was used to assess morpholino efficiency. Sting morpholino injection led to nonsense mediated decay of mRNA transcripts out to 5dpf. The Sting morpholino and the Ccr2, Pu.1(Cambier et al., 2014b), and Myd88 morpholinos (Bates et al., 2007) (see Key Resources Table) previously described were injected into the 1-4 cell stage of the developing embryo (Tobin et al., 2010).

#### Quantitative Real-time PCR (qRT-PCR)

Total RNA was isolated from pools of 20-40 larvae as previously described (Clay et al., 2007) and described herein, using TRIzol Reagent (Life Technologies), followed by chloroform precipitation. Isolated RNA was used to synthesize cDNA with Superscript III reverse transcriptase and oligo DT primers (ThermoFisher Scientific). Quantification of *ccl2, ifnΦ1, ifnΦ2, and ifnΦ3* RNA levels were determined using SYBR green PCR Master Mix (Applied Biosystems) on an ABI Prism 7300 Real-Time PCR System (Applied Biosystems) using the following primer pairs; *ccl2*: 5′GTCTGGTGCTCTTCGCTTTC3′ and 5′TGCAGAGAAGATGCGTCGTA3′, *ifnΦ1*: 5′TTAATACACGCAAAGATGAGAACTC3′ and 5′GCCAAGCCATTCGCAAGTAG3′, *ifnΦ2*: 5′CCTCTTTGCCAACGACAGTT3′ and 5′CGGTTCCTTGAGCTCTCATC3′, *ifnΦ3*: 5′GAGGATCAGGTTACTGGTGT3′ and 5′GTTCATGATGCATGTGCTGTA3′. Average values of technical triplicates of each biological replicate were plotted. Data were normalized to *β-actin* for ΔΔCt analysis using the following primer pair for *β-actin:* 5′AGAGGGAAATCGTGCGTGAC3′ and 5′CAATAGTGATGACCTGGCCGT3′ (Ramirez-Carrozzi et al., 2009).

#### Infectivity Assay

2 dpf larvae were infected via the hindbrain ventricle with an average of 0.8 bacteria per injection as previously described (Cambier et al., 2014b). Fish harboring 1-3 bacteria for some experiments or 1 bacterium for others were identified at 5 hr post infection by confocal microscopy. These infected fish were then evaluated at 5 dpi, or every 24 hr following infection, and were scored as infected or uninfected, based on the presence or absence of fluorescent bacteria.

#### CCL2 In Situ Hybridization

In situ hybridization was performed as previously described (Clay et al., 2007) and described herein: Zebrafish *ccl2* (ENSDARG00000041835) was cloned from adult pooled cDNA constructed from isolating RNA from homogenized adult tissues using Trizol (ThermoFisher), chloroform extraction and purification using RNeasy mini kit (QIAGEN). Superscript III reverse transcriptase (ThermoFisher) was used to make cDNA and the following primer pair 5′GTCAGCTAGGATCCATGAGGCCGTCCTGCATCC3′ and 5′GTCAGCTATCTAGATTAGGCGCTGTCACCAGAG3′ was used to clone zebrafish *ccl2*. *ccl2* cDNA was then cloned into the pCS2+ plasmid (A gift from Marc Kirschner, Addgene plasmid #17095), the plasmid was then linearized with the restriction factor HindIII (Thermofisher) and in vitro antisense RNA was synthesized with the T7 Megascript kit (Thermofisher) using DIG RNA labeling mix (Sigma) to make the antisense RNA in situ probe. Mm infected fish were then overdosed in tricaine and fixed overnight in 4% paraformaldehyde and then dehydrated by storage at −20C overnight in methanol. Fish were then rehydrated in PBS with 0.1% Tween 20 (PBST) and digested in 10μg/ml Proteinase K (Thermofisher) for 30min at room temperature. Fish were then refixed in 4% paraformaldehyde, washed in PBST and then hybridized with the antisense probe at 65C for 3hours. Fish were washed in PBST and then incubated with blocking reagent (PBST, 5% sheep serum (Sigma) and 2 mg/ml BSA (Sigma)) for 2hrs at room temperature. Fish were then incubated with anti-DIG-AP antibody (Sigma) at 1:5000 in blocking reagent overnight at 4C. Fish were then washed with PBST and developed with BM-purple (Sigma). Fish were then stored in glycerol and imaged.

#### Infection of Human Alveolar Macrophages

On the day of infection Mm wild-type and Δ*pks15* growing in Middlebrook 7H9 medium were centrifuged at 2900 g for 10min and resuspended in RPMI 1640 containing 10% FCS. Clumps were disrupted by passing the bacilli through a 25-gauge needle 6-8 times and the sample was centrifuged at 100 (x)g for 3 min to remove any remaining clumps. To assess the adequacy of dispersion and to determine the MOI, macrophages were infected with varying amounts of resuspended Mm wild-type and PGL-deficient for 2hrs. Extracellular bacteria were washed off, and cells were fixed with 2% paraformaldehyde for 10mins. Macrophage nuclei were counterstained with 10 μg/ml of Hoechst 33258 (Sigma). The percentage of infected cells and the number of bacilli per cell were determined by fluorescent microscopy (Olympus IX51, Olympus Europa GmbH, Germany) for each donor, as previously described (Gleeson et al., 2016, O’Leary et al., 2011, O’Sullivan et al., 2007, O’Leary et al., 2014). Based on this result alveolar macrophages were infected at an estimated MOI of 1-10 bacilli. At 1hr post-infection supernatants were harvested for CCL2 (MCP-1) assay.

#### MesoScale Discovery Chemokine (CCL2 (MCP1)) Assay

Human MCP-1 chemokine kit (Meso Scale Discovery®, Maryland, USA) was used as per manufacturers’ instructions, briefly samples, standards and controls were added at 25 μL per well. Detection antibody was added at 25 μL per well, 150 μL of the MSD Read Buffer was added to each well and the MSD plates were analyzed on the MSD Sector Imager 2400 plate reader. The raw data was measured as electrochemiluminescence signal (light) detected by photodetectors and analyzed using the Discovery Workbench 3.0 software (MSD). A 4-parameter logistic fit curve was generated for CCL2 (MCP1) using the standards and the concentration of each sample calculated.

### Quantification and Statistical Analysis

#### Statistics

The following statistical analyses were performed using Prism 5.01 (GraphPad): One-way ANOVA with Bonferroni’s post-test, Fisher’s exact test, Student’s unpaired t test, and one sample t test. Error bars represent standard error of mean. Post-test *P* values are as follows: ^∗^p < 0.05; ^∗∗^p < 0.01; ^∗∗∗^p < 0.001. The statistical tests used for each figure can be found in the corresponding figure legend. Where the *n* value is given and not represented graphically in the figure, *n* represents the number of zebrafish used for each experimental group.

## Author Contributions

C.J.C. and L.R. conceived, designed and analyzed the zebrafish experiments and C.J.C. performed them. C.J.C, S.M.O., M.P.O., J.K., and L.R. designed and analyzed the human experiments, and S.M.O performed them. C.J.C. and L.R. wrote the paper with input from S.M.O., M.P.O., and J.K.

## Supplemental Information

Table S1. related to Figure 4 The number of resident macrophages and monocytes responding to infection with 1–3 wild-type Mm or PDIM- Mm every 10 min from 1–11 hr post infection. Raw data used to make Figure 4A.

Table S2. related to Figure 4 Number of transfer events occurring during the first 4.5 days following infection with 1–3 bacteria. The number of fish imaged, fish remaining infected, number of total infected macrophages, and the number of transfer events recorded during each time-period of imaging are provided following infection with either wild-type or PGL^−^*M. marinum* (Mm). Raw data for Figure 4E.

Table S3. related to Figure 6 CCL2 production by human alveolar macorphages. A complete list of donor alveolar macrophages and their CCL2 production in response to LPS, wild-type Mm, and PGL^−^ Mm.

Movie S1. related to Figure 4 Transfer of green Wasabi-expressing wild-type Mm from red Mpeg1^+^-resident macrophage to blue Hoechst^+^ red Mpeg1^+^ monocyte. Imaged every 10 min.

Movie S2. related to Figure 4 Green Mpeg1^+^ cell infected with red TdTomato-expressing PGL^−^ Mm. Surface rendering done by setting a threshold for the red fluorescence using Imaris. Imaged every 10 min.

## References

[bib1] Aggad D., Mazel M., Boudinot P., Mogensen K.E., Hamming O.J., Hartmann R., Kotenko S., Herbomel P., Lutfalla G., Levraud J.P. (2009). The two groups of zebrafish virus-induced interferons signal via distinct receptors with specific and shared chains. J. Immunol..

[bib65] Antonelli L.R.V., Gigliotti Rothfuchs A., Gonçalves R., Roffê E., Cheever A.W., Bafica A. (2010). Intranasal Poly-IC treatment exacerbates tuberculosis in mice through the pulmonary recruitment of a pathogen-permissive monocyte/macrophage population. Journal of Clinical Investigation.

[bib2] Aston C., Rom W.N., Talbot A.T., Reibman J. (1998). Early inhibition of mycobacterial growth by human alveolar macrophages is not due to nitric oxide. Am. J. Respir. Crit. Care Med..

[bib3] Athman J.J., Wang Y., McDonald D.J., Boom W.H., Harding C.V., Wearsch P.A. (2015). Bacterial Membrane Vesicles Mediate the Release of Mycobacterium tuberculosis Lipoglycans and Lipoproteins from Infected Macrophages. J. Immunol..

[bib4] Bates J.H., Potts W.E., Lewis M. (1965). Epidemiology of Primary Tuberculosis in an Industrial School. N. Engl. J. Med..

[bib5] Bates J.M., Akerlund J., Mittge E., Guillemin K. (2007). Intestinal alkaline phosphatase detoxifies lipopolysaccharide and prevents inflammation in zebrafish in response to the gut microbiota. Cell Host Microbe.

[bib6] Berg R.D., Levitte S., O’Sullivan M.P., O’Leary S.M., Cambier C.J., Cameron J., Takaki K.K., Moens C.B., Tobin D.M., Keane J., Ramakrishnan L. (2016). Lysosomal Disorders Drive Susceptibility to Tuberculosis by Compromising Macrophage Migration. Cell.

[bib7] Bhatnagar S., Schorey J.S. (2007). Exosomes released from infected macrophages contain Mycobacterium avium glycopeptidolipids and are proinflammatory. J. Biol. Chem..

[bib8] Brannon M.K., Davis J.M., Mathias J.R., Hall C.J., Emerson J.C., Crosier P.S., Huttenlocher A., Ramakrishnan L., Moskowitz S.M. (2009). Pseudomonas aeruginosa Type III secretion system interacts with phagocytes to modulate systemic infection of zebrafish embryos. Cell. Microbiol..

[bib9] Cambier C.J., Falkow S., Ramakrishnan L. (2014). Host evasion and exploitation schemes of Mycobacterium tuberculosis. Cell.

[bib10] Cambier C.J., Takaki K.K., Larson R.P., Hernandez R.E., Tobin D.M., Urdahl K.B., Cosma C.L., Ramakrishnan L. (2014). Mycobacteria manipulate macrophage recruitment through coordinated use of membrane lipids. Nature.

[bib11] Casano A.M., Peri F. (2015). Microglia: multitasking specialists of the brain. Dev. Cell.

[bib12] Cepok S., Schreiber H., Hoffmann S., Zhou D., Neuhaus O., von Geldern G., Hochgesand S., Nessler S., Rothhammer V., Lang M. (2009). Enhancement of chemokine expression by interferon beta therapy in patients with multiple sclerosis. Arch. Neurol..

[bib13] Chen, H., Sun, H., You, F., Sun, W., Zhou, X., Chen, L., Yang, J., Wang, Y., Tang, H., Guan, Y., et al. (2011). Activation of STAT6 by STING Is Critical for Antiviral Innate Immunity. *147*, 436–446. 10.1016/j.cell.2011.09.022 22000020

[bib14] Clay H., Davis J.M., Beery D., Huttenlocher A., Lyons S.E., Ramakrishnan L. (2007). Dichotomous role of the macrophage in early Mycobacterium marinum infection of the zebrafish. Cell Host Microbe.

[bib15] Comas I., Chakravartti J., Small P.M., Galagan J., Niemann S., Kremer K., Ernst J.D., Gagneux S. (2010). Human T cell epitopes of Mycobacterium tuberculosis are evolutionarily hyperconserved. Nat. Genet..

[bib16] Conrady C.D., Zheng M., Mandal N.A., van Rooijen N., Carr D.J.J. (2013). IFN-α-driven CCL2 production recruits inflammatory monocytes to infection site in mice. Mucosal Immunol..

[bib17] Curto M., Reali C., Palmieri G., Scintu F., Schivo M.L., Sogos V., Marcialis M.A., Ennas M.G., Schwarz H., Pozzi G., Gremo F. (2004). Inhibition of cytokines expression in human microglia infected by virulent and non-virulent mycobacteria. Neurochem. Int..

[bib18] Davis J.M., Ramakrishnan L. (2009). The role of the granuloma in expansion and dissemination of early tuberculous infection. Cell.

[bib19] Dey B., Dey R.J., Cheung L.S., Pokkali S., Guo H., Lee J.-H., Bishai W.R. (2015). A bacterial cyclic dinucleotide activates the cytosolic surveillance pathway and mediates innate resistance to tuberculosis. Nat. Med..

[bib20] Epelman S., Lavine K.J., Randolph G.J. (2014). Origin and functions of tissue macrophages. Immunity.

[bib21] Gagneux S., DeRiemer K., Van T., Kato-Maeda M., de Jong B.C., Narayanan S., Nicol M., Niemann S., Kremer K., Gutierrez M.C. (2006). Variable host-pathogen compatibility in Mycobacterium tuberculosis. Proc. Natl. Acad. Sci. USA.

[bib22] Ge R., Zhou Y., Peng R., Wang R., Li M., Zhang Y., Zheng C., Wang C. (2015). Conservation of the STING-Mediated Cytosolic DNA Sensing Pathway in Zebrafish. J. Virol..

[bib23] Gleeson L.E., Sheedy F.J., Palsson-McDermott E.M., Triglia D., O’Leary S.M., O’Sullivan M.P., O’Neill L.A.J., Keane J. (2016). Cutting Edge: Mycobacterium tuberculosis Induces Aerobic Glycolysis in Human Alveolar Macrophages That Is Required for Control of Intracellular Bacillary Replication. J. Immunol..

[bib24] Gordon S., Plüddemann A., Martinez Estrada F. (2014). Macrophage heterogeneity in tissues: phenotypic diversity and functions. Immunol. Rev..

[bib25] Hall C., Flores M.V., Storm T., Crosier K., Crosier P. (2007). The zebrafish lysozyme C promoter drives myeloid-specific expression in transgenic fish. BMC Dev. Biol..

[bib26] Herbomel P., Thisse B., Thisse C. (2001). Zebrafish early macrophages colonize cephalic mesenchyme and developing brain, retina, and epidermis through a M-CSF receptor-dependent invasive process. Dev. Biol..

[bib27] Hirsch C.S., Ellner J.J., Russell D.G., Rich E.A. (1994). Complement receptor-mediated uptake and tumor necrosis factor-alpha-mediated growth inhibition of Mycobacterium tuberculosis by human alveolar macrophages. J. Immunol..

[bib28] Hocking W.G., Golde D.W. (1979). The pulmonary-alveolar macrophage (second of two parts). N. Engl. J. Med..

[bib29] Keane J., Remold H.G., Kornfeld H. (2000). Virulent Mycobacterium tuberculosis strains evade apoptosis of infected alveolar macrophages. J. Immunol..

[bib30] Lauvau G., Chorro L., Spaulding E., Soudja S.M. (2014). Inflammatory monocyte effector mechanisms. Cell. Immunol..

[bib31] Lesokhin A.M., Hohl T.M., Kitano S., Cortez C., Hirschhorn-Cymerman D., Avogadri F., Rizzuto G.A., Lazarus J.J., Pamer E.G., Houghton A.N. (2012). Monocytic CCR2(+) myeloid-derived suppressor cells promote immune escape by limiting activated CD8 T-cell infiltration into the tumor microenvironment. Cancer Res..

[bib32] Madigan C.A., Cambier C.J., Kelly-Scumpia K.M., Scumpia P.O., Cheng T.-Y., Zailaa J., Bloom B.R., Moody D.B., Smale S.T., Sagasti A. (2017). A macrophage response to *Mycobacterium leprae* phenolic glycolipid initiates nerve damage in leprosy. Cell.

[bib33] Manzanillo P.S., Shiloh M.U., Portnoy D.A., Cox J.S. (2012). Mycobacterium tuberculosis activates the DNA-dependent cytosolic surveillance pathway within macrophages. Cell Host Microbe.

[bib34] Martinez F.O., Gordon S. (2014). The M1 and M2 paradigm of macrophage activation: time for reassessment. F1000Prime Rep..

[bib35] Murray P.J., Allen J.E., Biswas S.K., Fisher E.A., Gilroy D.W., Goerdt S., Gordon S., Hamilton J.A., Ivashkiv L.B., Lawrence T. (2014). Macrophage activation and polarization: nomenclature and experimental guidelines. Immunity.

[bib36] O’Leary S., O’Sullivan M.P., Keane J. (2011). IL-10 blocks phagosome maturation in mycobacterium tuberculosis-infected human macrophages. Am. J. Respir. Cell Mol. Biol..

[bib37] O’Leary S.M., Coleman M.M., Chew W.M., Morrow C., McLaughlin A.M., Gleeson L.E., O’Sullivan M.P., Keane J. (2014). Cigarette smoking impairs human pulmonary immunity to Mycobacterium tuberculosis. Am. J. Respir. Crit. Care Med..

[bib38] O’Sullivan M.P., O’Leary S., Kelly D.M., Keane J. (2007). A caspase-independent pathway mediates macrophage cell death in response to Mycobacterium tuberculosis infection. Infect. Immun..

[bib39] Onwueme K.C., Vos C.J., Zurita J., Ferreras J.A., Quadri L.E.N. (2005). The dimycocerosate ester polyketide virulence factors of mycobacteria. Prog. Lipid Res..

[bib40] Pagán A.J., Yang C.-T., Cameron J., Swaim L.E., Ellett F., Lieschke G.J., Ramakrishnan L. (2015). Myeloid Growth Factors Promote Resistance to Mycobacterial Infection by Curtailing Granuloma Necrosis through Macrophage Replenishment. Cell Host Microbe.

[bib41] Pang J.M., Layre E., Sweet L., Sherrid A., Moody D.B., Ojha A., Sherman D.R. (2012). The polyketide Pks1 contributes to biofilm formation in Mycobacterium tuberculosis. J. Bacteriol..

[bib42] Parichy D.M., Ransom D.G., Paw B., Zon L.I., Johnson S.L. (2000). An orthologue of the kit-related gene fms is required for development of neural crest-derived xanthophores and a subpopulation of adult melanocytes in the zebrafish, Danio rerio. Development.

[bib43] Ramirez-Carrozzi V.R., Braas D., Bhatt D.M., Cheng C.S., Hong C., Doty K.R., Black J.C., Hoffmann A., Carey M., Smale S.T. (2009). A unifying model for the selective regulation of inducible transcription by CpG islands and nucleosome remodeling. Cell.

[bib44] Rhoades E., Hsu F., Torrelles J.B., Turk J., Chatterjee D., Russell D.G. (2003). Identification and macrophage-activating activity of glycolipids released from intracellular Mycobacterium bovis BCG. Mol. Microbiol..

[bib45] Rich E.A., Torres M., Sada E., Finegan C.K., Hamilton B.D., Toossi Z. (1997). Mycobacterium tuberculosis (MTB)-stimulated production of nitric oxide by human alveolar macrophages and relationship of nitric oxide production to growth inhibition of MTB. Tuber. Lung Dis..

[bib46] Roca F.J., Ramakrishnan L. (2013). TNF dually mediates resistance and susceptibility to mycobacteria via mitochondrial reactive oxygen species. Cell.

[bib66] Rutledge B.J., Rayburn H., Rosenberg R., North R.J., Gladue R.P., Corless C.L., Rollins B.J. (1995). High level monocyte chemoattractant protein-1 expression in transgenic mice increases their susceptibility to intracellular pathogens. Journal of Immunology (Baltimore, Md. : 1950).

[bib47] Samstein M., Schreiber H.A., Leiner I.M., Sušac B., Glickman M.S., Pamer E.G., Lanzavecchia A. (2013). Essential yet limited role for CCR2^+^ inflammatory monocytes during Mycobacterium tuberculosis-specific T cell priming. eLife.

[bib48] Serbina N.V., Jia T., Hohl T.M., Pamer E.G. (2008). Monocyte-mediated defense against microbial pathogens. Annu. Rev. Immunol..

[bib49] Shi C., Pamer E.G. (2011). Monocyte recruitment during infection and inflammation. Nat. Rev. Immunol..

[bib50] Siméone R., Sayes F., Song O., Gröschel M.I., Brodin P., Brosch R., Majlessi L. (2015). Cytosolic access of Mycobacterium tuberculosis: critical impact of phagosomal acidification control and demonstration of occurrence in vivo. PLoS Pathog..

[bib51] Spanos J.P., Hsu N.-J., Jacobs M. (2015). Microglia are crucial regulators of neuro-immunity during central nervous system tuberculosis. Front. Cell. Neurosci..

[bib52] Srivastava S., Ernst J.D., Desvignes L. (2014). Beyond macrophages: the diversity of mononuclear cells in tuberculosis. Immunol. Rev..

[bib53] Steele S., Radlinski L., Taft-Benz S., Brunton J., Kawula T.H. (2016). Trogocytosis-associated cell to cell spread of intracellular bacterial pathogens. eLife.

[bib54] Takaki K., Cosma C.L., Troll M.A., Ramakrishnan L. (2012). An in vivo platform for rapid high-throughput antitubercular drug discovery. Cell Rep..

[bib55] Takaki K., Davis J.M., Winglee K., Ramakrishnan L. (2013). Evaluation of the pathogenesis and treatment of Mycobacterium marinum infection in zebrafish. Nat. Protoc..

[bib56] Tobin, D.M., Vary, J.C., Jr, Ray, J.P., Walsh, G.S., Dunstan, S.J., Bang, N.D., Hagge, D.A., Khadge, S., King, M.-C., Hawn, T.R., et al. (2010). The lta4h Locus Modulates Susceptibility to Mycobacterial Infection in Zebrafish and Humans. *140*, 717–730. 10.1016/j.cell.2010.02.013 PMC2907082 20211140

[bib57] Urdahl K.B. (2014). Understanding and overcoming the barriers to T cell-mediated immunity against tuberculosis. Semin. Immunol..

[bib58] van Zyl-Smit R.N., Binder A., Meldau R., Semple P.L., Evans A., Smith P., Bateman E.D., Dheda K. (2014). Cigarette smoke impairs cytokine responses and BCG containment in alveolar macrophages. Thorax.

[bib59] Volkman H.E., Clay H., Beery D., Chang J.C.W., Sherman D.R., Ramakrishnan L. (2004). Tuberculous granuloma formation is enhanced by a mycobacterium virulence determinant. PLoS Biol..

[bib60] Volkman H.E., Pozos T.C., Zheng J., Davis J.M., Rawls J.F., Ramakrishnan L. (2010). Tuberculous granuloma induction via interaction of a bacterial secreted protein with host epithelium. Science.

[bib64] Wang N., Tytell J.D., Ingber D.E. (2009). Mechanotransduction at a distance: mechanically coupling the extracellular matrix with the nucleus. Nature Reviews Molecular Cell Biology.

[bib61] Wells W.F., Ratcliffe H.L., Grumb C. (1948). On the mechanics of droplet nuclei infection; quantitative experimental air-borne tuberculosis in rabbits. Am. J. Hyg..

[bib62] Wolf A.J., Linas B., Trevejo-Nuñez G.J., Kincaid E., Tamura T., Takatsu K., Ernst J.D. (2007). Mycobacterium tuberculosis infects dendritic cells with high frequency and impairs their function in vivo. J. Immunol..

[bib63] Yang C.-T., Cambier C.J., Davis J.M., Hall C.J., Crosier P.S., Ramakrishnan L. (2012). Neutrophils exert protection in the early tuberculous granuloma by oxidative killing of mycobacteria phagocytosed from infected macrophages. Cell Host Microbe.

