# Supplementary figures and images for: Phenolic Glycolipid Facilitates Mycobacterial Escape from Microbicidal Tissue-Resident Macrophages
Source: Immunity. 2017 Sep 19;47(3):552–565.e4. doi: 10.1016/j.immuni.2017.08.003 (PMC5610147; doi:10.1016/j.immuni.2017.08.003)

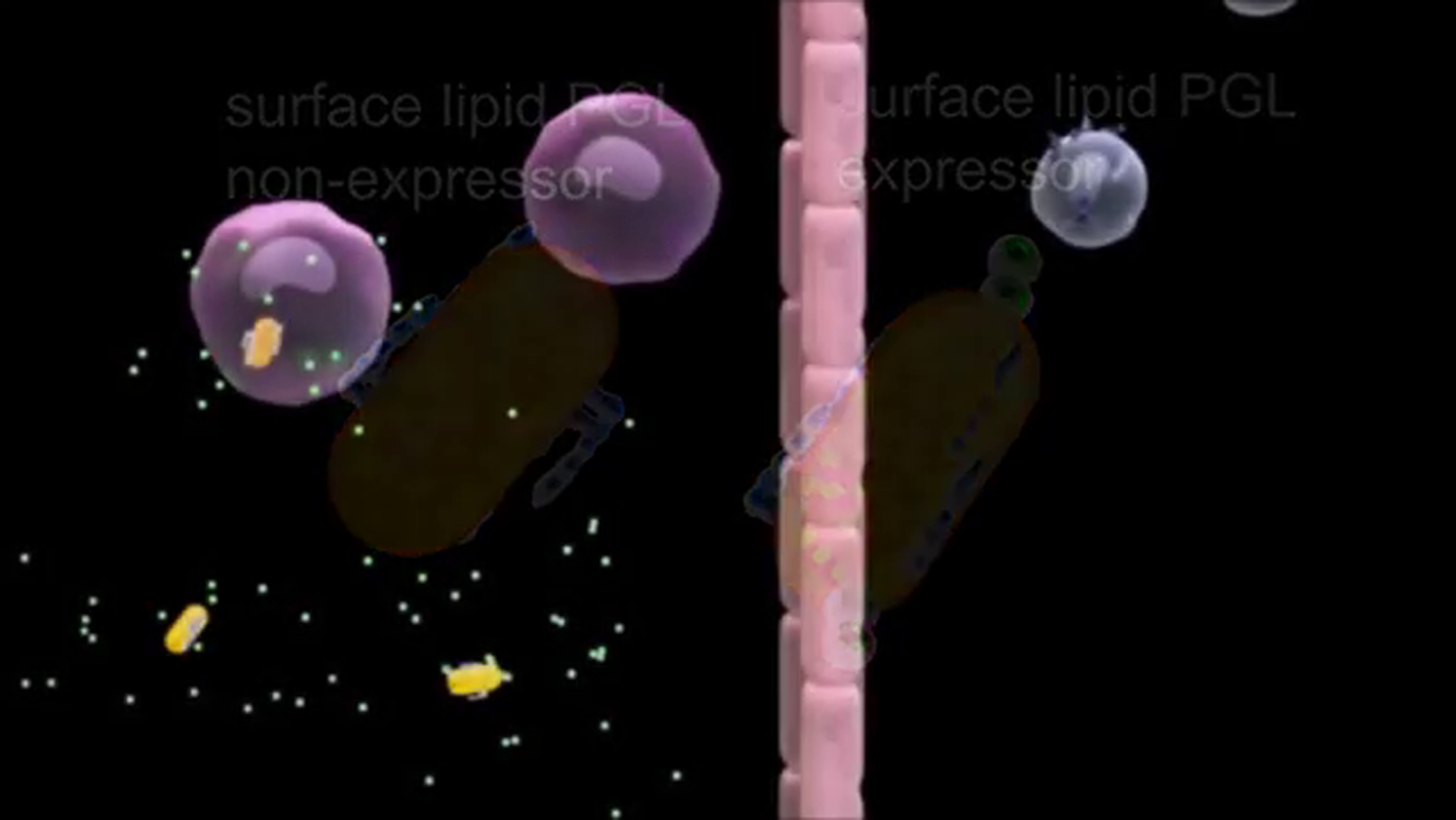

Supplement: Supplementary file 1 [file mmc6.jpg]

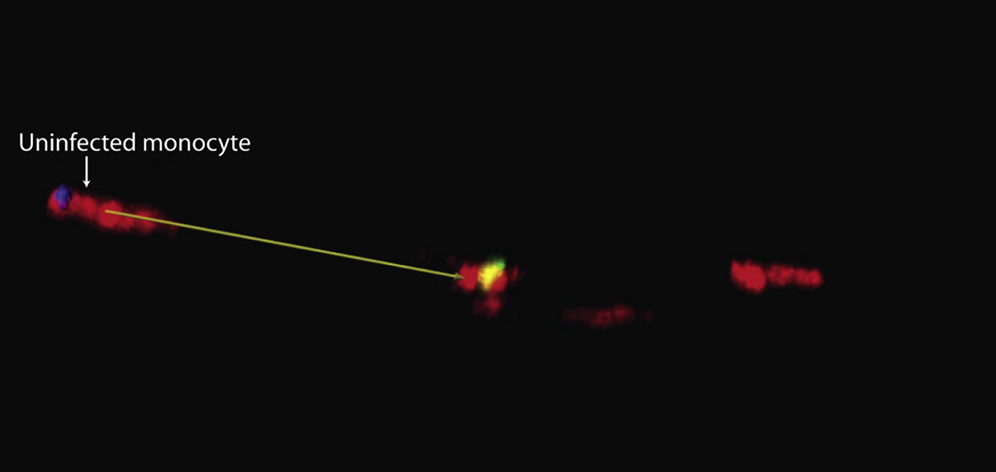

Supplement: Movie S1. related to Figure 4 — Transfer of green Wasabi-expressing wild-type Mm from red Mpeg1+-resident macrophage to blue Hoechst+ red Mpeg1+ monocyte. Imaged every 10 min. [file mmc4.jpg]

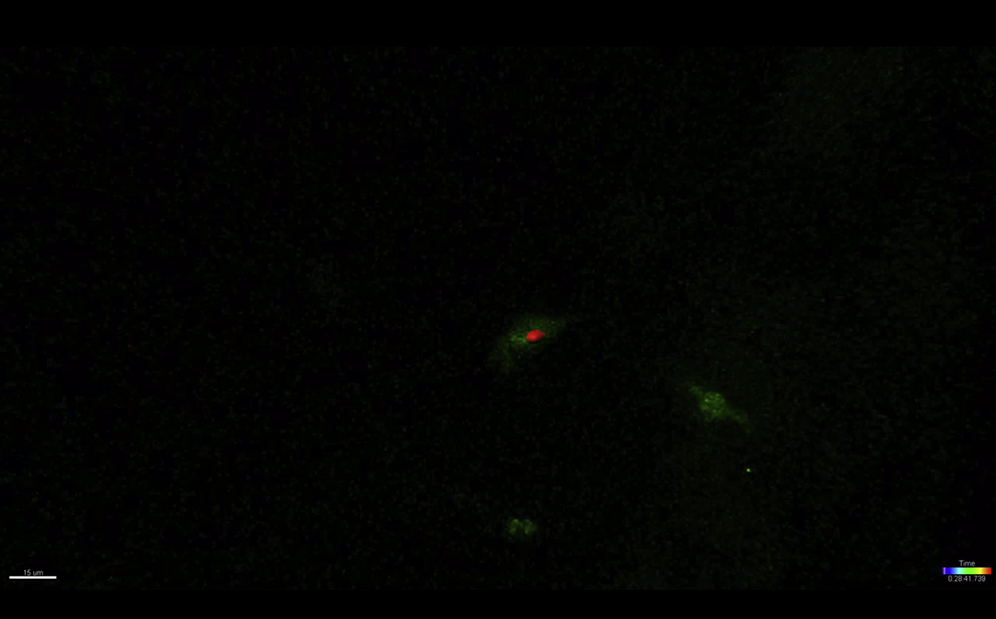

Supplement: Movie S2. related to Figure 4 — Green Mpeg1+ cell infected with red TdTomato-expressing PGL− Mm. Surface rendering done by setting a threshold for the red fluorescence using Imaris. Imaged every 10 min. [file mmc5.jpg]
